# Case Report: Clinicopathological features and literature review of a case of lymphoepithelioma-like intrahepatic cholangiocarcinoma

**DOI:** 10.3389/fonc.2026.1881159

**Published:** 2026-07-15

**Authors:** Ruiping Cao, Xiuling Hu, Song Huang, Hongzhu Yan

**Affiliations:** 1 Department of Pathology, Seventh People’s Hospital of Shanghai University of Traditional Chinese Medicine, Shanghai, China; 2 Department of Radiology, Seventh People’s Hospital of Shanghai University of Traditional Chinese Medicine, Shanghai, China

**Keywords:** adenoid differentiation, EBV-associated cholangiocarcinoma, lymphoepithelioma-like intrahepatic cholangiocarcinoma, pathological diagnosis, pseudocapsule

## Abstract

**Background:**

Lymphoepithelioma-like intrahepatic cholangiocarcinoma (LEL-ICC) is a rare, Epstein-Barr virus (EBV)-associated subtype of intrahepatic cholangiocarcinoma (ICC). With fewer than 150 cases reported globally, its comprehensive clinicopathological spectrum and optimal management remain incompletely understood, necessitating updated, systematic evaluations.

**Case presentation:**

This report describes a case of LEL-ICC incidentally discovered in a 71-year-old female patient. Imaging and gross pathological examinations revealed that the tumor had well-defined borders and a distinct pseudocapsule, a feature rarely emphasized in the description of such lesions. Histological examination showed that the tumor consisted of moderately to highly differentiated glandular structures, with a stroma composed of follicular lymphoid tissue. The tumor cells expressed cholangiocarcinoma markers (cytokeratin 7 and cytokeratin 19) but did not express markers of hepatocellular origin or those commonly associated with extrahepatic metastases. Combined immunohistochemical analysis revealed a wild-type expression pattern for p53, a Ki-67 proliferation index of approximately 10%, and positivity for EB virus-encoded small RNA (EBER); these findings collectively confirmed the final diagnosis.

**Conclusion:**

The lesion in this case presented with focal adenoid differentiation and an intact pseudocapsule, further enriching our understanding of the morphological characteristics of intrahepatic cholangiocarcinoma (ICC) with lymphoepithelial differentiation. This article also reviews the relevant literature and summarizes the etiology, diagnostic criteria, differential diagnosis, and treatment options for this disease. This study further confirms that EBER testing for hepatitis-associated tumors can effectively reduce misdiagnosis and holds significant clinical value.

## Introduction

Lymphoepithelioma-like carcinoma (LELC) is a rare malignant neoplasm characterized by undifferentiated or poorly differentiated epithelial cells accompanied by dense and prominent lymphocytic and plasmacytic infiltration, which is morphologically similar to undifferentiated nasopharyngeal carcinoma (1–3). First reported in the nasopharynx, this tumor has subsequently been documented in multiple organs, including the lung, stomach, salivary gland, thymus, and liver (4–6). Lymphoepithelial-like hepatocellular carcinoma (LEL-HCC) and Lymphoepithelioma-like intrahepatic cholangiocarcinoma (LEL-ICC)are two subtypes of lymphoepithelial-like carcinoma of the liver; the latter is a relatively rare subtype of ICC (7–9).

Since first reported by Hsu et al. in 1996, LEL-ICC has been strongly associated with Epstein-Barr virus (EBV) infection, and most cases are positive for EBER by *in situ* hybridization (10–13). Epidemiologically, LEL-ICC occurs predominantly in middle-aged to elderly Asian females, often in the absence of chronic viral hepatitis or liver cirrhosis (14–16). Most patients are asymptomatic, with lesions detected incidentally; preoperative imaging is nonspecific and frequently misdiagnosed as hepatocellular carcinoma (10, 17–19).

Typical histopathological features of LEL-ICC include undifferentiated or poorly differentiated cells arranged in syncytial or nests, abundant lymphoplasmacytic infiltration in the stroma, and the absence of adenoid differentiation or its presence only focally (20–22).If the tumor exhibits a complete pseudocapsule accompanied by distinct adenoid structures, this presentation is relatively rare and is easily misdiagnosed as conventional intrahepatic cholangiocarcinoma or a capsulated hepatic tumor (23, 24). This article reports a case of LEL-ICC exhibiting the aforementioned morphological features and discusses the findings in light of the relevant literature.

## Case description

A 71-year-old woman was incidentally found to have a liver mass during a routine physical examination. The patient had no history of hepatitis B or C infection, and all preoperative laboratory tests, including liver function tests, were normal. Serum tumor marker levels, including carcinoembryonic antigen (CEA), carbohydrate antigen 125 (CA125), carbohydrate antigen 19-9 (CA19-9), and alpha-fetoprotein (AFP), were also unelevated. Contrast-enhanced magnetic resonance imaging (MRI) revealed a nodule measuring 1.9 × 1.8 × 1.6 cm in segment II of the liver; the imaging findings were suggestive of hepatocellular carcinoma. After assessing the patient’s health status, she underwent laparoscopic segmental hepatectomy.

### Imaging findings

Further evaluation of the lesion demonstrated slight hypointensity on T1-weighted imaging (T1WI) and slight hyperintensity on T2-weighted imaging (T2WI), with restricted diffusion on diffusion-weighted imaging (DWI). Imaging findings include: contrast-enhanced MRI showed marked arterial phase hyperenhancement with rapid washout, accompanied by a distinct pseudocapsule on delayed-phase images (**Figure 1**). This specific combination of dynamic enhancement patterns closely mimicked the classic radiologic phenotype of hepatocellular carcinoma, leading to the initial misdiagnosis.

**Figure 1:**
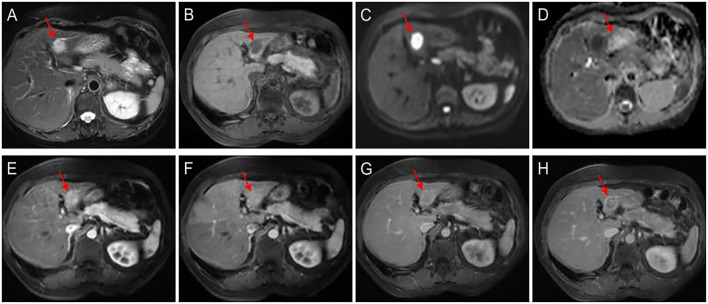
Magnetic resonance imaging (MRI) features of the hepatic nodule in segment II. **(A)** T2WI shows homogeneous, slight hyperintensity. **(B)** T1WI shows homogeneous hypointensity. **(C)** DWI demonstrates high signal intensity, corresponding to **(D)** low signal intensity on the apparent diffusion coefficient (ADC) map, indicating restricted diffusion (ADC value: 0.654 × 10^-3^ mm²/s). **(E)** Early arterial phase imaging reveals marked homogeneous hyperenhancement. **(F)** Late arterial and **(G)** portal venous phase images show isointensity relative to the surrounding liver parenchyma, with the latter demonstrating ring-like enhancement of a pseudocapsule. **(H)** Delayed-phase imaging demonstrates relative hypointensity (indicating washout), accompanied by persistent ring-like enhancement of the pseudocapsule.

### Gross findings

Gross evaluation of the resected specimen revealed a predominantly well-circumscribed, firm, gray-white nodule measuring 1.9 × 1.8 × 1.6 cm within the hepatic parenchyma. While a distinct pseudocapsule clearly demarcated most of the tumor from the adjacent non-neoplastic liver tissue, a focal area of infiltrative growth was macroscopically evident (**Figure 2**).

**Figure 2:**
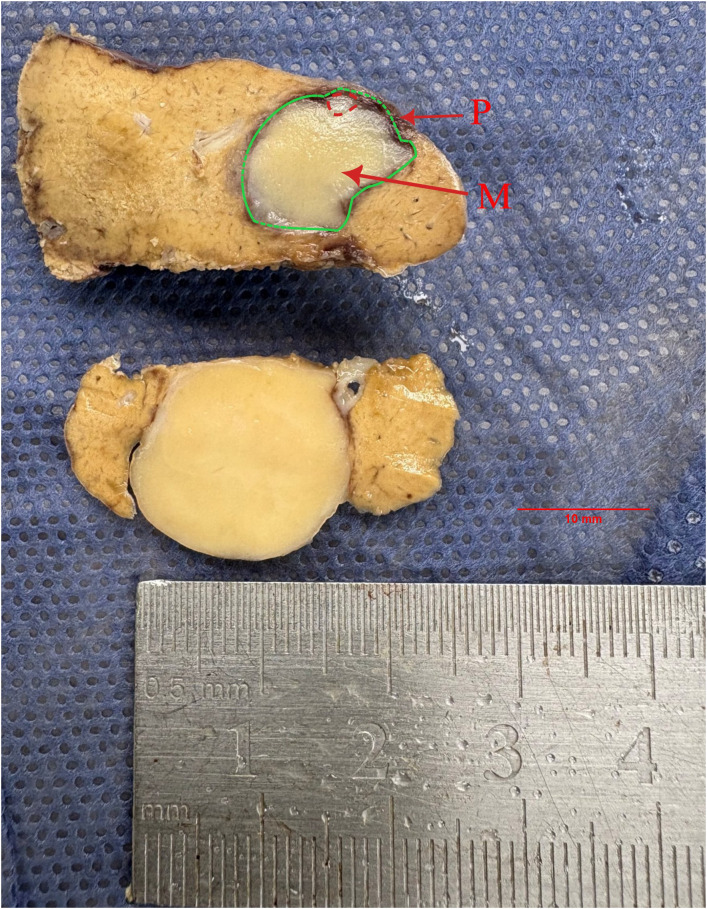
Gross appearance of the resected LEL-ICC specimen. M, main tumor mass. The distinct pseudocapsule, indicated by P and the green dotted line, demarcates the lesion from the adjacent hepatic parenchyma. The red dotted circle highlights a focal area of infiltrative growth.

### Microscopic findings

Microscopically, low-power evaluation of the hematoxylin and eosin (H&E)-stained sections revealed a predominantly pushing tumor border, consistent with the macroscopic pseudocapsule, despite focal areas of infiltrative growth (**Figure 3A**). At higher magnification, the tumor was composed of nests of atypical epithelial cells. In some areas, these cells exhibited syncytial growth with indistinct cell borders, vesicular nuclei, fine chromatin, and identifiable mitotic figures (**Figure 3B**). Focally, prominent moderately to well-formed adenoid structures were observed (**Figure 3C**). There is diffuse infiltration of a large number of lymphocytes and plasma cells within the tumor stroma, often forming distinct lymphoid follicles. No fibrotic reaction was observed, nor were there any clear signs of vascular or perineural invasion.

**Figure 3:**
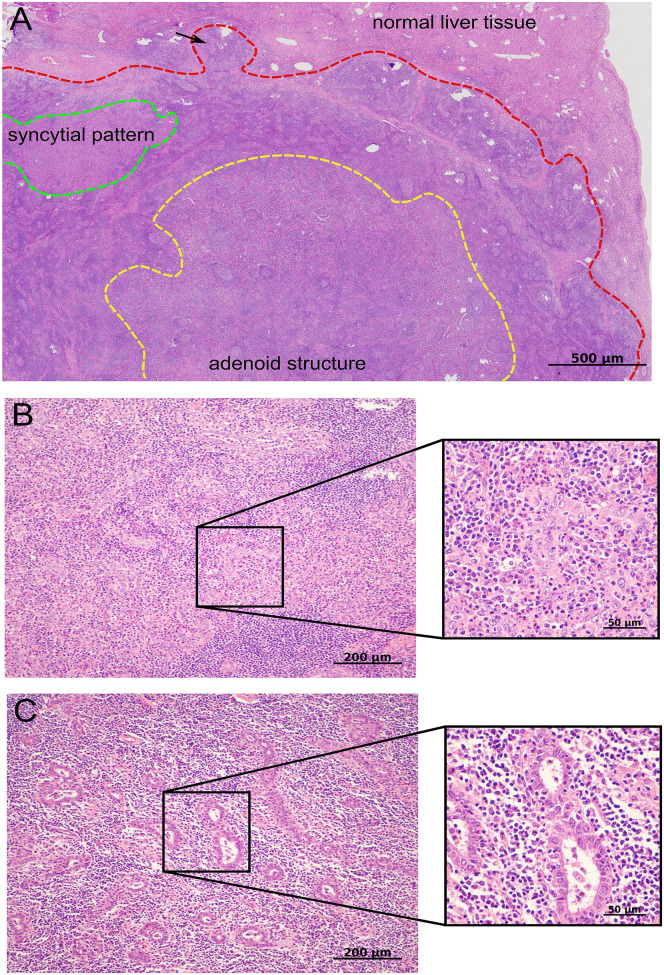
Histopathological features of the LEL-ICC. **(A)** Low-power view showing a predominantly pushing tumor border with a focal area of infiltrative growth (H&E, 20×, black arrows). **(B)** High-power view of the syncytial growth pattern, highlighting indistinct cell borders and vesicular nuclei (H&E, left, 100×; right, 400×). **(C)** High-power view demonstrating well-formed adenoid structures (H&E, left, 100×; right, 400×).

### Immunohistochemistry and EBER ISH

Immunohistochemical analysis showed that the tumor cells were positive for CK7 and CK19 (**Figures 4A, B**), supporting their cholangiocarcinoma-like differentiation. In contrast, markers derived from hepatocytes (HepPar-1, arginase-1, and GPC3), markers of intestinal differentiation (CK20, CDX2), and markers of other common primary sites (TTF1, PAX8, GATA3, and P40) were all negative. Notably, EBER *in situ* hybridization revealed diffuse, strongly positive nuclear staining in the tumor cells (**Figure 4C**). Combined with histological findings, the tumor was diagnosed as EBV-positive LEL-ICC. Based on histomorphological characteristics, the final diagnosis was EBV-positive LEL-ICC. The tumor measured 1.9 × 1.8 × 1.6 cm, with no evidence of definite microvascular invasion, and the surgical margins were negative. The patient underwent laparoscopic hepatic segmentectomy; no lymph node specimens were submitted for examination. According to the 8th edition of the American Joint Committee on Cancer (AJCC) tumor TNM classification, the pathological stage of this case was pT1aN_X_M_X_.

**Figure 4:**
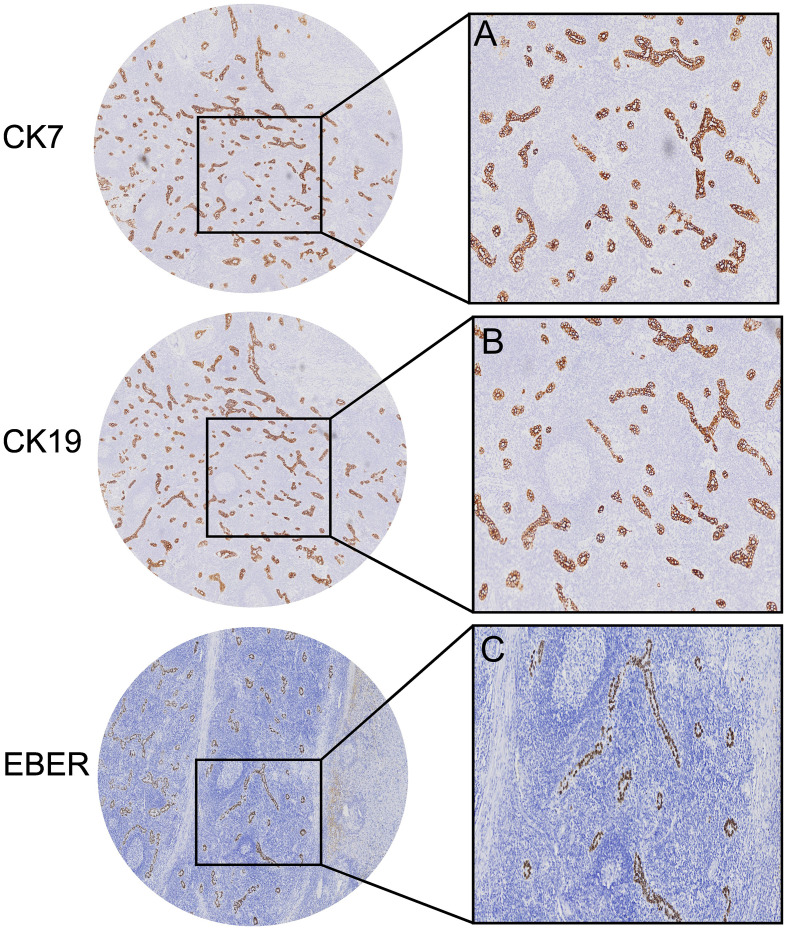
Immunohistochemical and EBER *in situ* hybridization profiles. **(A)** CK7 and **(B)** CK19 expression in tumor cells (left 40×; right 200×), supporting biliary origin. **(C)** EBER *in situ* hybridization demonstrating diffuse and robust nuclear positivity (left 40×; right 200×), confirming the association with EBV.

## Discussion

This case presents an EBV-associated LEL-ICC characterized by focal adenoid differentiation (with adenoid structures accounting for approximately 20% of the tumor) and an intact pseudocapsule; both of these are relatively rare morphological features (3–6). Although typical LEL-ICC consists of undifferentiated syncytial cells, occasional reports of focal adenoid differentiation have been documented in the literature (12–14). Therefore, this case does not represent a completely novel and unique phenotype, but rather enriches the currently known morphological spectrum of LEL-ICC. This indicates the necessity of performing EBER *in situ* hybridization testing on all liver tumors exhibiting a prominent lympho-plasma cell matrix (regardless of the degree of epithelial differentiation); its broader clinical-pathological significance will be discussed below.

### Histopathological and immunophenotypic features

This highlights a key diagnostic pitfall: in heavily inflamed hepatic tumors, the presence of mature adenoid structures should not serve as grounds for excluding an EBV-driven process, thereby avoiding misclassification as lymphocyte-rich/inflammatory-type intrahepatic cholangiocarcinoma or hepatocellular carcinoma (4, 21). Strong CK7 and CK19 expression confirms biliary differentiation (15–17), while diffuse nuclear EBER positivity verifies EBV association, seen in approximately 70–80% of cases (18–20). The dense lymphoplasmacytic infiltrate rich in T cells and B lymphocytes, accompanied by prominent lymphoid follicle formation, reflects the characteristic immune-rich microenvironment, which is thought to underlie the tumor’s indolent behavior and relatively favorable prognosis (21–23, 25).

### Pseudocapsule: A key diagnostic and prognostic clue

A well-defined pseudocapsule was observed on imaging, gross examination, and microscopy. This feature provides an important diagnostic clue in the present case, but its weight in prognostic assessment should be interpreted with caution. In limited case series of LEL-ICC, observations have suggested that when a tumor forms a distinct pseudocapsule, it tends to exhibit a pushing border, lacks a prominent desmoplastic reaction, shows relatively low intratumoral heterogeneity, and may have a lower incidence of vascular invasion; some related studies have also indicated a trend toward prolonged survival in such patients (4–6, 26). However, given the rarity of LEL-ICC and the fact that the existing evidence is largely derived from retrospective case series, these associations have not been independently validated and should not yet be regarded as universal prognostic predictors. This morphological feature can, to a certain extent, aid in the distinction from conventional mass-forming intrahepatic cholangiocarcinoma, which typically lacks an intact pseudocapsule and displays a highly infiltrative border (16–18). As a combined imaging and pathological assessment indicator, even though focal infiltrative areas were seen microscopically in this case, the presence of a well-circumscribed pseudocapsule suggests that the tumor may have a relatively indolent biological behavior. However, this interpretation is mainly based on small-sample descriptions and, in part, on experience extrapolated from other Epstein-Barr virus (EBV)-associated tumors (e.g., nasopharyngeal carcinoma, lymphoepithelioma-like carcinoma); its value as an independent favorable prognostic marker remains to be confirmed by larger-scale, LEL-ICC-specific studies (6, 21).

### EBV pathogenesis and genomic characteristics

According to the literature, EBV drives LEL-ICC tumorigenesis through epigenetic dysregulation, TME remodeling, and LMP1/EBNA1-mediated NF-κB activation, which upregulates PD-L1 and recruits the characteristic lymphoid infiltrate (9, 19–21, 27). Genomically, TERT promoter and TP53 mutations occur in 60–75% of EBV-positive cases (7–9). It is worth noting that the patient in this case demonstrated a wild-type expression pattern for p53 on immunohistochemistry. Since the tumor was detected at an early stage and had been completely resected, comprehensive genetic testing could not be performed. In summary, this case confirms that EBV-induced malignant transformation is not limited to poorly differentiated tumors but may, conversely, exhibit atypical adenoid differentiation features (9, 21, 28).

### Differential diagnosis

It is crucial to accurately distinguish LEL-ICC from morphologically similar lesions. In this case, the lesion exhibits well-differentiated glandular structures; therefore, the primary differential diagnosis is lymphocyte-rich/inflammatory intrahepatic cholangiocarcinoma. Clinical symptoms of Lymphocyte-rich intrahepatic cholangiocarcinoma (LR-ICC) may include fever, jaundice, pruritus, recurrent cholangitis, and weight loss. Patients often have a history of long-standing biliary tract damage, and CA19–9 and CEA levels are typically markedly elevated. Imaging studies may reveal delayed enhancement. The two conditions are primarily distinguished through histopathological examination. The differential diagnosis between the two is primarily based on three key factors: tumor cell morphology, the type of stromal reaction, and the status of EB virus infection. LR-ICC typically exhibits tubular or adenoid differentiation, with relatively mild cellular atypia and well-defined cell borders; its lymphocytic infiltration density is lower than that of LEL-ICC, and it is often accompanied by a fibrotic stromal reaction. In the lesions of the conventional type of intrahepatic cholangiocarcinoma, neutrophils and histiocytes can also be detected (7–9) (**Table 1**).

**Table 1:** Key distinguishing features between LEL-ICC and LR-ICC.

Feature	Lymphoepithelioma-like ICC (LEL-ICC)	Lymphocyte-rich ICC (LR-ICC)
Tumor cell & histological morphology	Cells predominantly syncytial, with only focal adenoid differentiation	Typically tubular or glandular differentiation
Density of stromal lymphocytic infiltration	Markedly abundant, frequently outnumbering tumor cells	Lymphocytic infiltration present, but not to the extent seen in LEL-ICC
Types of infiltrating cells	Predominantly lymphoplasmacytic; lymphoid follicle formation may occur	Predominantly lymphocytic; neutrophils and histiocytes may also be present
Stromal reaction pattern	Desmoplastic stromal reaction typically absent	Desmoplastic stromal reaction frequently present
EBV association	EBER *in situ* hybridization positive in the vast majority of cases (~75%)	Relatively low; only a minority of cases are EBV-associated

Another condition requiring differential diagnosis is lymphoepithelial-like hepatocellular carcinoma (LEL-HCC). The vast majority of patients with LEL-HCC have a history of viral hepatitis-related cirrhosis and present with symptoms associated with cirrhosis, such as ascites, splenomegaly, hypersplenism, and gastrointestinal bleeding; AFP levels may be markedly elevated; during the contrast-enhancement phase of imaging, the typical “rapid uptake and rapid washout” pattern of HCC may be observed; differentiation between the two is primarily based on histopathology. Although both share histological features of undifferentiated cancer cells accompanied by extensive lymphocytic infiltration, they exhibit significant differences in immunophenotype, EB virus infection rates, clinical and pathological characteristics, and prognosis (11–13, 19) (**Table 2**).

**Table 2:** Key distinguishing features between LEL-ICC and LEL-HCC.

Feature	LEL-ICC	LEL-HCC
Tumor cell & histological morphology	Cells predominantly syncytial, with only focal adenoid differentiation	Syncytial areas may be accompanied by trabecular/pseudoglandular patterns (HCC architecture)
Immunohistochemistry	CK7+, CK19+, HepPar-1−, Arginase-1−	HepPar-1+, Arginase-1+, CK7−/CK19−
EBER in situ hybridization	Positive in approximately 75% of cases	Rarely positive (~<10%)

Finally, distinguishing LEL-ICC from LELC (such as lesions originating in the nasopharynx or lung) requires consideration of both the patient’s clinical information and imaging findings. In this case, the biliary immunophenotype (CK7+, CK19+), combined with negative extrahepatic markers (TTF-1−, p40−, CDX2−) and the absence of abnormalities on systemic imaging studies, strongly confirms that this tumor is a primary liver lesion.

### Therapeutic strategies and clinical management

Surgical resection is the cornerstone of curative treatment for LEL-ICC, and the 5-year overall survival rate for patients who undergo R0 resection can reach 65% to 70% or higher (2, 5, 6). The patient in this case underwent complete R0 resection; due to the tumor’s high degree of differentiation and slow progression, our patient did not receive any postoperative treatment. Four months after surgery, the patient is currently feeling well. For unresectable, recurrent, or metastatic lesions, clinical treatment primarily relies on immune checkpoint inhibitors. A study by Zheng LN et al. demonstrated that patients with early-stage EBV-LELCC have a favorable prognosis following curative surgery; even among patients with advanced EBV-LELCC, those with stage IV disease can achieve a 7-year survival rate after receiving combination therapy involving multiple anticancer modalities, including surgery, interventional therapy, radiation therapy, chemotherapy, targeted therapy, and immunotherapy (3). In a report by Zhu YT et al., LEL-ICC was characterized as a typical EBV-associated “hot tumor,” exhibiting high expression of programmed death ligand-1 (PD-L1) and abundant lymphocytic infiltration within the lesions. Therefore, disease can be well controlled using treatment regimens based on anti-programmed death receptor-1 (PD-1)/PD-L1 therapy (20, 29).

For smaller recurrent lesions, local ablation therapy can also achieve long-term disease control (15). Long-term follow-up is crucial for early detection of recurrence and the timely initiation of immunotherapy or local salvage therapy.

### Prognosis and prognostic factors

Compared with traditional nodular intrahepatic cholangiocarcinoma, LEL-ICC has a relatively better prognosis and is one of the slowest-progressing subtypes of malignant tumors of the biliary tract (2, 5, 13). Literature indicates that the 5-year overall survival rate after complete resection reaches 70%–80% for LEL-ICC, whereas that for conventional ICC typically remains below 20% (2, 5). Even patients presenting with advanced disease tend to exhibit substantially longer survival trajectories than those with non-LEL counterparts (3, 5).

Current literature reports that several key clinical and pathological features have been identified as indicators of a favorable prognosis, including EB virus positivity, R0 resection, absence of lymph node metastasis, presence of an intact pseudocapsule, moderate to high differentiation, abundant CD8-positive T-cell infiltration within the tumor, and absence of vascular and perineural invasion (2–6, 20–24). In this case, the patient exhibits nearly all of the aforementioned favorable prognostic features. As this surgical procedure has been performed for a relatively short period, long-term follow-up data are currently unavailable, and long-term follow-up is required.

### Limitations and future perspectives

Although this study is limited by its single-case design and the lack of comprehensive molecular profiling, the findings point to several important directions for future research in this area. Primarily, there is an urgent need to establish standardized diagnostic criteria for gland-rich and pseudocapsule-bearing morphological variants of LEL-ICC to prevent clinical misclassification (3–7). In tandem, validating circulating EBV-DNA as a non-invasive biomarker could significantly refine early diagnosis and post-treatment surveillance (21). Furthermore, while our patient was amenable to curative resection, managing advanced or refractory disease remains a formidable challenge; thus, exploring dual immune checkpoint blockade and novel targeted strategies is essential to improve outcomes in these patients (24). Ultimately, clarifying the underlying molecular dichotomies between EBV-positive and EBV-negative subgroups will be crucial to enable precision stratification and personalized therapy for this rare malignancy (30).

## Conclusion

This case expands the known morphological spectrum of EBV-associated LEL-ICC by presenting the complete constellation of markedly well-differentiated adenoid structures and a distinct gross pseudocapsule. Crucially, recognizing this variant underscores the necessity of integrating virological status (EBV evaluation) with routine histopathology to prevent misclassification as conventional ICC, thereby guiding appropriate clinical management. In summary, large-scale, multicenter studies are still needed to comprehensively map the molecular profiles of these rare, slow-growing malignancies, refine prognostic biomarkers, and optimize targeted treatment regimens.

## Data Availability

The original contributions presented in the study are included in the article/Supplementary Material. Further inquiries can be directed to the corresponding author.
